# Acute effects of whole-body vibration on unilateral isometric knee extensors maximal torque and fatigability during an intermittent endurance task in adult males

**DOI:** 10.1016/j.heliyon.2024.e35822

**Published:** 2024-08-10

**Authors:** Francesca Greco, Gian Pietro Emerenziani, Katia Folino, Marco Spadafora, Loretta Francesca Cosco, Carolina Muscoli, Paolo Sgrò, Federico Quinzi

**Affiliations:** a Department of Movement, Human and Health Sciences, University of Rome “Foro Italico”, 00135, Rome, Italy; b Department of Clinical and Experimental Medicine, University of Catanzaro “Magna Græcia”, 88100, Catanzaro, Italy; c Department of Movement Sciences and Wellness, University Parthenope, 80133, Naples, Italy; d Department of Health Science, Institute of Research for Food Safety & Health (IRC-FSH), "Magna Græcia" University of Catanzaro, 88100, Catanzaro, Italy

**Keywords:** Muscle endurance, Fatigue, Torque signal, Vibration

## Abstract

Whole-body vibration (WBV) has been employed for performance-enhancing purposes. WBV may positively affect muscular endurance and its underlying neural mechanisms due to an enhanced muscular blood circulation and oxygen uptake. However, the effects of WBV on endurance-related torque signal complexity have been understudied. This study aims to investigate the acute effects of WBV on i) maximal isometric torque production; ii) isometric knee extensors fatigability and iii) torque signal complexity during an isometric endurance task.

Thirty adult males performed an isometric intermittent endurance protocol on their dominant lower limb after performing: static half squat with WBV (WBV), static half squat without WBV (HS), and no exercise protocol (CC). For each repetition the maximal torque was identified. The maximal torque of the first repetition was identified as the PeakT. The Mean torque (MTorque) and fatigue index (pFatigue) were calculated as the mean and the percentage decay in torque across the entire set of eighteen repetitions (MTorque_0–100 %_, pFatigue_0–100 %_), and across shorter blocks of six repetitions (MTorque_0–33 %_, pFatigue_0–33 %_; MTorque_34–66 %_, pFatigue_34–66 %_, and MTorque_67–100 %_, pFatigue_67–100 %_). Torque fluctuations were analysed computing Sample Entropy (SampEn) and the coefficient of variation (CV).

PeakT was significantly higher in CC than in WBV (p < 0.01) and in HS (p < 0.01). PeakT was significantly higher in HS than in WB (p < 0.05). MTorque_0–100 %_, MTorque_0–33 %_, MTorque_34–66 %,_ and MTorque_67–100 %_ were significantly higher in CC than in WBV (all p-values <0.01) and in HS (p < 0.01). MTorque_67–100 %_ was significantly higher in HS than in WB (p = 0.049). pFatigue_34–66 %_ was significantly higher in WBV than in CC (p < 0.05) whereas pFatigue_67–100 %_ was significantly higher in CC than in WB (p < 0.01) and in HS (p < 0.01). No effect of condition was observed for SampEn and CV.

Acute WBV does not lead to beneficial effects on maximal torque production and isometric knee extensors fatigability. These acute detrimental effects may be related to long-term WBV-related adaptations.

## 1 Introduction

Muscular fitness, which incorporates the functional components of muscular strength, endurance, and power, is widely recognised as a critical aspect of good physical function. Specifically, lower limb muscular fitness, mainly evaluated by means of knee extensors assessments tend to decrease more rapidly than upper limb [1]. Remarkably, lower limb muscular fitness is involved in most of everyday tasks (e.g., walking, stair-climbing), thereby representing a key health-related component to be preserved throughout life [1,2]. Whole-body vibration (WBV) training has long been recognised as an alternative exercise modality for sport and health-enhancing purposes [3]. Specifically, it has been proposed to resemble adaptive responses like those obtained from muscle-strengthening exercises [4]. Indeed, one of the first applications of WBV was to investigate its potential effects on lower-body maximal force production and power [5]. Regarding the acute effects of WBV on lower-body force production, several investigations have been conducted [[6], [7], [8]]. The acute exposure of WBV prior to static [[6], [7], [8]] or dynamic tasks reported inconclusive results in terms of muscular strength and power [9]. Specifically, a reduction in voluntary maximal isometric knee extensor force was found after both 30 Hz [[6], [7]] and 50 Hz [8] of WBV exposure. These debated findings may depend on methodological discrepancies as well as on the application of different vibration parameters (i.e., frequency, duration, amplitude) that may have affected muscles’ force-generating capacity differently [3,5,10]. A previous study focused on the effects of WBV on mean force production during a brief intermittent isometric endurance protocol [11]. That study showed a detrimental effect of WBV on mean force production. However, in that study, other mechanical and physiological parameters were neglected. Moreover, the protocol adopted by the authors was relatively short and may have been inadequate to induce acute neuromuscular fatigue [11]. Therefore, effects of WBV on muscular endurance remain largely uninvestigated and relevant mechanisms of WBV are still unclear. Several mechanisms have been proposed on the possible acute effects of WBV. Since WBV exposure elicits a tonic reflex contraction and stimulates anabolic hormones production it may influence skeletal muscle fibres length and speed thereby enhancing muscular strength and power [5,12,13]. Moreover, WBV, due to enhanced muscular blood circulation and oxygen uptake, may positively affect muscular endurance [14,15]. Therefore, this study focuses on the acute effects of WBV on knee extensors i) maximal isometric torque production and ii) knee extensor endurance while performing an isometric endurance task. To gain further insight into the mechanisms of action of WBV on muscular endurance, torque fluctuations were also investigated. We expected that the exposure to WBV may increase muscle force production, due to its positive effect on neuromuscular system, and muscular endurance, due to the WBV-induced peripheral blood responses. It was hypothesized that an increased metabolic power induced by WBV could lead to muscular improvements in terms of endurance.

## 2 Methods

### 2.1 Study design

Participants performed one pre-testing session and three testing sessions at the laboratory of Physical Exercise and Sport Sciences at the “Magna Græcia” University of Catanzaro. Each test session was interspersed by 72 h to avoid possible fatigue and muscle soreness [2]. In the pre-test session, participants familiarised with the experimental procedure (i.e. WBV protocol and isometric force protocol) and anthropometric measures and dominant lower limb were recorded. Lower limb dominance was assessed by asking participants which foot they would use to kick a stationary ball [16]. In each test session, participants performed an isometric leg extension protocol on the dominant lower limb in three different experimental sessions in a random order: static half squat with WBV (WBV), static half squat without WBV (HS), and no exercise protocol (CC). The experimental procedures were the same of those reported in a previous study [11]. The Declaration of Helsinki was followed to conduct all the experimental procedures. Study protocol was approved by the regional ethical committee (approval number n. 122/2021).

### 2.2 Participants

An a priori sample size estimation analysis (G-Power 3.1.9.2) showed that twenty-eight participants were sufficient to achieve a statistical power of β = 0.80 (effect size computed from mean force observed in Greco et al. [11]. Thirty males completed the whole experimental protocol (Mean ± Standard Deviation (SD); Age: 41.2 ± 9.4 years; BMI: 26.3 ± 4.8 kg/m^2^). Inclusion criteria were males, age range 26–60 years, not currently involved in any type of resistance training activity no previous experience with WBV, any physical activity contraindication assessed with the physical activity readiness questionnaire (PAR-Q+) [17]. Exclusion criteria were any self-reported cognitive impairment, an history of lower limb injury in the twelve months preceding the study. Participants were informed of the aims, risks and benefits of the study and signed the written informed consent to participate in the study.

### 2.3 Data collection

#### 2.3.1 Anthropometric measures

Height, body mass and body compositions parameters were evaluated as previously described [11]. Specifically, participants’ skeletal muscle mass (32.6 ± 3.3 kg) and percentage of fat mass (27.1 ± 6.1 %) were estimated. Physical activity levels (PAL) in three domains (i.e., activity at work, travel to and from places, and recreational activities) were assessed using the Global Physical Activity Questionnaire (G-PAQ) [18]. Our participants reported a PAL of 891.2 ± 926.9 METs-min per week.

#### 2.3.2 Isometric force protocol

A standardized warm-up consisting in 7 min of pedalling at 50 rpm on a cycling ergometer (Ergoselect, ergoline GmbH, Bitz, Germany) followed by 3 min of knee extensors stretching exercises was performed before the isometric endurance protocol. Then, participants randomly performed three different experimental conditions:

o half squat with WBV (WBV): participants were asked to stand on a Pro Evolve vibrating platform (DKN, USA) in static half squat (90° of knee flexion) for five consecutive exercise bouts each lasting 60 s at 30 Hz (This protocol was adapted from the seminal work of Bosco et al. [19].
o half squat without WBV (HS): participants were in the same position of the WBV condition The vibrating platform was switched off.
o no exercise protocol (CC): control condition where no exercise protocol was performed.

Details of the three experimental conditions are reported in Greco et al. [11].

**Fig. 1:**
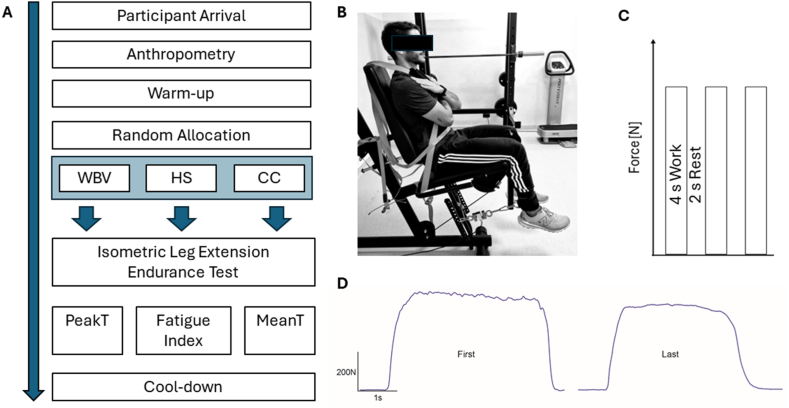
Schematic representation of the experimental set-up. A) Flowchart of the study protocol; B) Participant's positioning for the intermittent isometric endurance protocol; C) Description of the intermittent isometric leg extension endurance protocol; D) typical example of the Force signal recorded from the first and last repetition of the intermittent isometric endurance protocol from a representative participant. Acronyms: WBV, half squat plus WBV; HS, half squat without WBV; CC, no exercise protocol.

Participants were then asked to complete the isometric leg extension endurance protocol as showed in Fig. 1, panel B (Nextline Leg extension; Visa Sport, Marcellinara, Italy). The data acquisition setup was done using the MuscleLab™ 6000 system as reported elsewhere [11,20]. Participants were asked to extend the knee of their dominant limb as forcefully as possible against an immovable load for eighteen maximal efforts held for 4 s with 2 s of rest between each repetition. Specifically, they were instructed to contract their knee extensor muscles following using a visual countdown (Fig. 1, panel C). The whole endurance protocol was a modified version of a previous one [21]. For all participants, the fatiguing task lasted 106 s. Their knee-shank angle was set at 90° (with 0° representing knee full extension) and their trunk-thigh angle of 90°. Adjustable belts crossing participants’ shoulders and pelvis were used to minimize body movements during the task. Knee extensors moment arm was measured from the lateral knee condyle to the ankle strap (Fitgriff GmbH, Bergheim, Austria). At the end of the endurance protocol, participants performed lower limb stretching exercises. A representation of study protocol is reported in Fig. 1, panel A.

#### 2.3.3 Force data collection

Force data were recorded by using the MuscleLab™ 6000 Ergotest Innovation system and the software (MuscleLab v. 10.213.98.5188, Porsgrunn, Norway). The sampling frequency of the load cell was 200 Hz using a single data interface. Force signals were multiplied for the moment arm to obtain Torque. As a measure of maximal isometric torque production, peak torque (PeakT) was detected as the highest value on the torque-time curve for each contraction.

To investigate knee extensors endurance performance the mean torque and fatigue index were computed. Specifically, the mean torque was calculated averaging the PeakT across eighteen repetitions (MTorque_0–100 %_). To investigate the time-course of knee extensors fatigue, mean torque was also calculated considering the PeakT expressed in the first block of six repetitions (Mtorque_0–33 %_), in the second block of six repetitions (Mtorque_34–66 %_) and in the third block of six repetitions (Mtorque_67–100 %_).

The fatigue index was calculated as described in White et al. [21] namely, as the percentage of the difference between the last (PeakT_18_) and the first contraction (PeakT_1_) (Fig. 1, panel D) for the entire set of eighteen repetitions (pFatigue_0–33 %_) and for each of the blocks described above (pFatigue_0–33 %_; pFatigue_34–66 %_; pFatigue_67–100 %_). Specifically, the formula used to compute the Fatigue index is the following: [(PeakT_1_-PeakT_18_)/PeakT_1_] x 100.

To gain further insight into the mechanisms of action of WBV on muscular endurance, torque fluctuations were investigated computing the Sample Entropy (SampEn) as reported in Richman and Moorman [22] and the coefficient of variation (CV). SampEn an CV were computed for each participant and condition (CC, HS and WBV) for the first and for the eighteenth repetitions.

#### 2.3.4 Statistical analysis

Statistical analysis was carried out using IBM®SPSS statistics software version 23.0 (SPSS Inc., Chicago, IL, USA). The normal distribution of the dependent variables was tested using the Shapiro-Wilk test. Mauchly's sphericity test was used for each ANOVA model. Separate one-way ANOVA for repeated measures were performed to assess the effects of vibration stimuli on i) maximal isometric torque production (PeakT) and on ii) knee extensors endurance variables (MTorque_1-18_, MTorque_0–33 %_, MTorque_34–66 %,_ MTorque_67–100 %,_ pFatigue_1-18,_ pFatigue_0–33 %_; pFatigue_34–66 %_; pFatigue_67–100 %_). Bonferroni test was carried out as post hoc analyses and effect sizes were calculated with Cohen's d. The level of significance was set at p < 0.05.

*Discriminant analysis:* To gain insight into the factors that could account for the effectiveness of WBV on muscular endurance, participants were divided into responders and non-responders following a median split computed on the difference between the Fatigue Index after HS and WBV (Fatigue Index of HS minus Fatigue Index of WBV). To identify the anthropometrics variables that best predicted responders and non-responders to WBV a stepwise discriminant analysis was carried out including anthropometric (height) and body composition variables (body mass, skeletal muscle mass, percentage of fat mass).

*Signal complexity analysis:* SampEn and CV were submitted to a two-way RM-ANOVA with condition (CC, HS and WBV) and time (First vs Last repetition – CC1; CC18; HS1; HS18; WBV1; WBV18). Bonferroni corrected pairwise comparisons were carried out when main effects or interactions were observed.

## 3 Results

### 3.1 Maximal isometric torque production

**Fig. 2:**
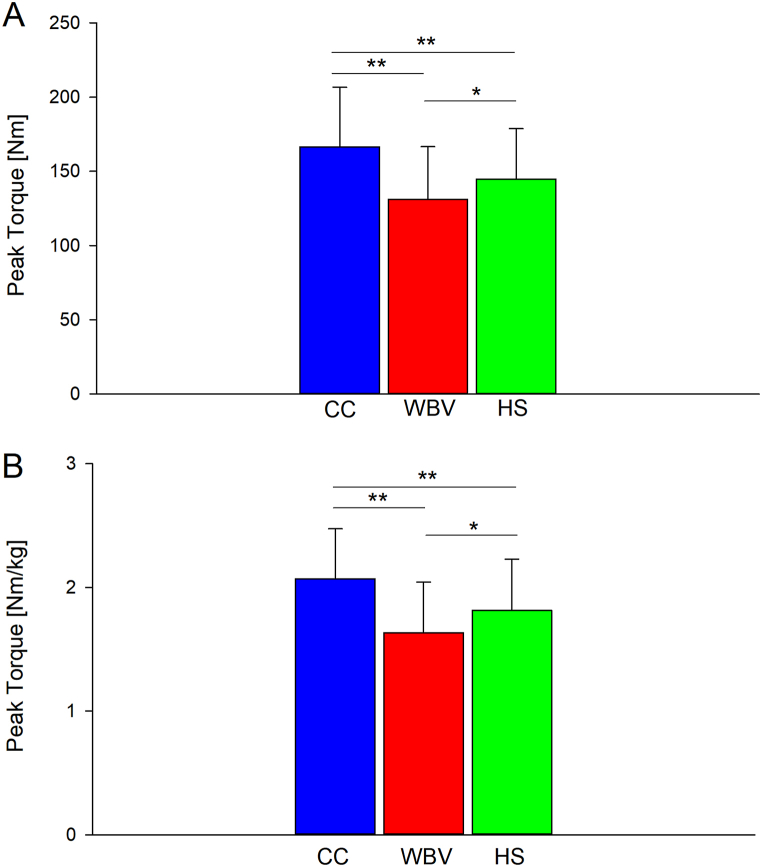
Results of maximal isometric torque production (PeakT). Panel A, PeakT values across conditions; panel B, Peak T normalized to body mass across conditions. Acronyms: CC, no exercise protocol; WBV, half squat plus WBV; HS, half squat without WBV *p<0.05;**p<*0.01.*

A significant main effect of condition was observed on PeakT (F_2,58_ = 27.956, p < 0.01, η^2^ = 0.491). Post hoc analysis showed that PeakT was significantly higher in CC (166.5 ± 40.1 Nm) than in WBV (131.1 ± 35.6 Nm, p < 0.01, d = 0.933) and in HS (144.8 ± 34.1 Nm, p < 0.01, d = 0.583). Moreover, PeakT was significantly higher in HS than in WBV (144.8 ± 34.1 Nm vs 131.1 ± 35.6 Nm, p < 0.05, d = 0.393) (Fig. 2, panel A). Peak torque data normalized to body mass are reported in Fig. 2, panel B.

### 3.2 Knee extensors endurance performance

**Fig. 3:**
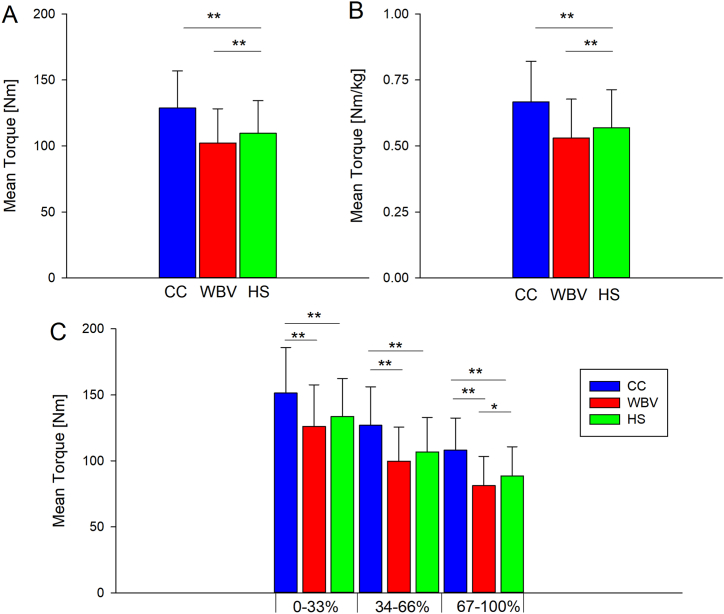
Results of Mean torque across the endurance task. Panel A, MTorque_0–100 %_ values across conditions; panel B, MTorque_0–100 %_ values normalized to body mass across conditions; panel C, MTorque values across eighteen repetitions in each condition. Acronyms: CC, no exercise protocol; WBV, half squat plus WBV; HS, half squat without WBV *p<0.05;**p<*0.01.*

A significant main effect of condition was observed on MTorque_0–100 %_ (F_2,58_ = 34.365, p < 0.01, η^2^ = 0.542). Post hoc analysis showed that MTorque_0–100 %_ was significantly higher in CC (128.8 ± 28.2 Nm) than in WBV (102.3 ± 25.9 Nm, p < 0.01, d = 0.978) and in HS (109.6 ± 24.8 Nm, p < 0.01, d = 0.723) (Fig. 3, panel A). Mean torque data normalized to body mass are reported in Fig. 3, panel B.

**Fig. 4:**
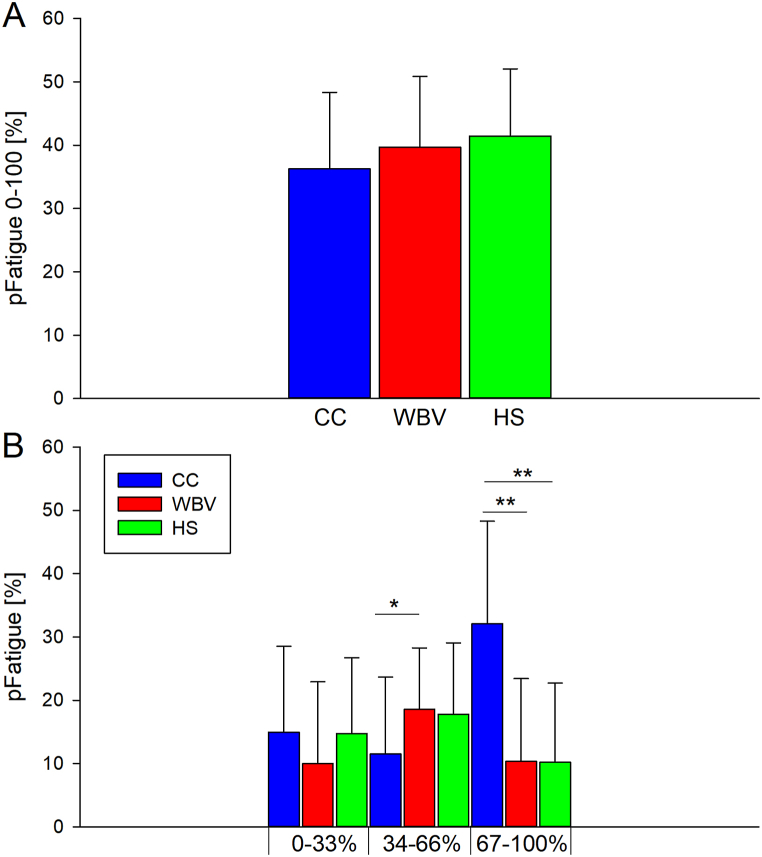
Results of pFatigue across the endurance task. Panel A, pFatigue_0–100 %_ values across conditions; panel B, pFatigue values across eighteen repetitions in each condition. Acronyms: CC, no exercise protocol; WBV, half squat plus WBV; HS, half squat without WBV *p<0.05; **p<*0.01.*

A significant main effect of condition was also observed on Mtorque_0–33 %_ (F_1.6,50.8_ = 21.957, p < 0.01, η^2^ = 0.431), MTorque_34–66 %_ (F_2,58_ = 28.430, p < 0.01, η^2^ = 0.495) and Mtorque_67–100 %_ (F_2,58_ = 41.605, p < 0.01, η^2^ = 0.589). Post hoc analysis showed that MTorque_0–33 %_ was significantly higher in CC (151.3 ± 34.6 Nm) than in WBV (126.1 ± 31.5 Nm, p < 0.01, d = 0.761) and in HS (133.6 ± 28.8 Nm, p < 0.01, d = 0.556) as well as MTorque_34–66 %_ (CC: 126.8 ± 29. 2 Nm; WB: 99.6 ± 25.8 Nm, p < 0.01, d = 0.987; HS: 106.8 ± 26.2 N m, p < 0.01, d = 0.721). (Fig. 3, panel C). Post hoc analysis showed that MTorque_67–100 %_ was significantly higher in CC (108.2 ± 24.3 Nm) than in WBV (81.2 ± 22.2 Nm, p < 0.01, d = 1.160) and in HS (88.6 ± 22.0 Nm, p < 0.01, d = 0.846). Moreover, MTorque_67–100 %_ was significantly higher in HS than in WBV (88.6 ± 22.0 Nm vs 81.2 ± 22.2 Nm, p < 0.05, d = 0.335) (Fig. 3, panel C). No significant effect of condition on pFatigue_0–100 %_ (F_2,58_ = 3.147, p = 0.05, η^2^ = 0.098) and on pFatigue_0–33 %_ (F_1.6,46.3_ = 2.685, p = 0.09, η^2^ = 0.085) was observed (Fig. 4, panel A and B). Instead, a significant main effect of condition was observed on pFatigue_34–66 %_ (F_2,58_ = 5.067, p < 0.01, η^2^ = 0.149) and on pFatigue_67–100 %_ (F_2,58_ = 40.847, p < 0.01, η^2^ = 5.585). Post hoc analysis showed that pFatigue_34–66 %_ was significantly higher in WBV (18.6 ± 9.7 %) than in CC (11.5 ± 12.2 %, p < 0.05, d = 0.644) and that pFatigue_67–100 %_ was significantly higher in CC (32.1 ± 16.2 %) than in WB (10.4 ± 13.1 %, p < 0.01, d = 1.473) and in HS (10.2 ± 12.6 %, p < 0.01, d = 1.509) (Fig. 4, panel B).

### 3.3 Discriminant analysis results

The stepwise discriminant function showed that the model was significant (Wilks Λ = 0.608; p = 0.004). The standardised canonical discriminant function coefficients showed that the variable able to discriminate between Responders and Non-responders were: percentage of fat mass (1.152), height (1.266) and skeletal muscle mass (−1.088). The ability of the model to correctly classify participants was 80 % both belonging to the Responders and Non-responders subgroups.

### 3.4 Signal complexity results

The RM-ANOVA carried out on SampEn revealed no significant effect of time (F_1,29_ = 0.820; p = 0.373, η^2^ = 0.027), condition (F_2,58_ = 1.061; p = 0.353, η^2^ = 0.035) and time by condition interaction (F_2,58_ = 2.706; p = 0.075, η^2^ = 0.085). Descriptive statistics of this variable across time and conditions are reported in Table 1. The RM-ANOVA carried out on the CV showed a significant effect of time (F_1,29_ = 15.57; p < 0.001, η^2^ = 0.349) with the eighteenth repetition showing larger CV than the first repetition (p < 0.001). No significant effect of condition (F_2,58_ = 0.110; p = 0.896, η^2^ = 0.004) and time by condition interaction (F_2,58_ = 0.045; p = 0.956, η^2^ = 0.002) were observed.

**Table 1:** Sample Entropy (SampEn) and Coefficient of Variation (CV) of the first and last repetitions across conditions.

	CC		HS		WBV	
	First	Last	First	Last	First	Last
SampEn	0.027(0.024)	0.019(0.012)	0.026(0.021)	0.033(0.047)	0.018(0.008)	0.032(0.046)
CV	0.255(0.095)	0.324(0.139)	0.244(0.106)	0.320(0.101)	0.249(0.093)	0.315(0.115)

CC, no exercise protocol; HS, half squat without WBV; WBV, half squat with WBV.

## 4 Discussion

This study aimed to evaluate i) maximal isometric torque production and ii) knee extensor endurance performance after WBV exposure. We expected that the exposure to WBV may increase muscle force production, due to its positive effect on neuromuscular system, and muscular endurance, due to the WBV-induced peripheral blood responses. Our results shows that WBV did not enhance knee extensors’ muscular performance during an acute exposure.

Previous investigations have also shown that acute exposure to WBV had no effects on the improvement of muscular isometric performance [7,8,11]. Despite the WBV used in the current investigation (half squat position at 90° of knee flexion at a vibration frequency of 30 Hz) has been shown to elicit the highest reflex response in vastus lateralis muscle [23], our results showed that acute exposure to WBV did not ameliorate muscle force production. Indeed, our results highlighted a higher peak torque production in CC than the other two test sessions (WBV and HS conditions). Moreover, maximal peak torque production was reduced after WBV exposure. These results align with previous investigations reporting no improvements on peak torque production after vibration stimulation [6,8,24]. Possibly, the contraction modality adopted in the present study may have masked the beneficial effects of WBV. Indeed, the reduced variation in muscle length during an isometric contraction compared to a stretch-shortening contraction may have concealed the effects of WBV [8,11]. Moreover, the WBV protocol adopted in the present study may also account for the lower knee extensors isometric peak torque after WBV exposure. Indeed, it could that the protocol proposed in the current investigation was too demanding for non-regularly exercising adults. Therefore, the effects of WBV may also depend by the protocol selected and by the participants’ training status.

The mean torque across the eighteen repetitions was higher in CC than in HS and in WBV. Moreover, when considering the three blocks, a steep decrease of mean torque was observed in WBV in the last block. Indeed, when analysing the last block (MTorque_67–100 %_), the mean torque was lower in the WBV compared to the HS condition. Therefore, WBV elicited worse performance in terms of mean torque production when compared to a conventional exercise protocol performed without vibration stimuli. However, it is worth noting that these apparently deleterious acute effects could facilitate long-term WBV-related adaptations. In contrast with these results, previous studies have reported no difference with the same exercise executed after WBV when analysing neuromuscular fatigue [25,26]. Specifically, central and peripheral mechanisms do not appear to differ between exercise with and without WBV irrespective of the origin of the induced fatigue [25,26]. It could be possible that our WBV protocol may have induced a larger neuromuscular fatigue compared to the other conditions (HS and CC). However, with the current setup, it cannot be established whether central or peripheral mechanisms account for the higher percentage of fatigue observed in the WBV condition.

Fatigue index has changed at block _34–66 %_ in the WBV condition despite no differences were found across the eighteen repetitions and during the first block (1^st^-6^th^ repetition). This may suggest an earlier decline of force production in the WBV condition compared to CC and HS. In the last block (13^th^-18^th^ repetition) of the isometric endurance protocol, participants reported a higher fatigue index (pFatigue_67–100 %_) in CC, compared to the other conditions. This may be due to the higher mean force during the entire testing session in CC than WBV and HS conditions. Therefore, in CC, participants produced higher force output across the eighteen repetitions thereby getting more tired. Despite some hypotheses have been reported, WBV application still need more investigations to analyse possible physiological mechanisms contributing to adaptive muscular responses after WBV exposure.

As regards the discriminant analysis, to the best of our knowledge, this is the first attempt to investigate possible predictors of the effects of WBV on muscular endurance. We showed that percentage of body fat, body height and skeletal muscle mass are suitable predictors to classify participants as responders or non-responders to WBV to increase endurance task performance. It can be speculated that, taken together, these factors may mediate the transmission of the vibratory stimulus to the knee extensors muscle and therefore influence the individual response to WBV stimuli. Further studies are needed in the future to confirm these preliminary results.

Concerning the signal complexity analysis, previous studies showed a decrease in torque complexity during fatiguing contractions [27]. Although the exact mechanisms accounting for the reduction in muscle torque complexity during fatiguing contraction are not fully understood at present, mechanical [28] and high density sEMG studies [29] posited that signal complexity may be related to the common synaptic input to muscle motor neurons. In the present study, we did not observe a decrease in the signal complexity (as reflected by the SampEn), this may be partly due to differences in the characteristics of the endurance protocol used in our study. In particular, in a previous study [27] a higher number of contractions was performed compared to our study. Indeed, compared to the study of Pethick et al. [27], in the present study shorter contraction duration were employed. These characteristics may have blunted the reduction in signal complexity observed in previous studies. As a final remark, our participants were older compared to those of Pethick et al. [27]. Previous studies showed that older adults display higher performance during endurance task compared to younger ones [30,31]. Noteworthy, a reduction in torque complexity has been reported in older adults compared to younger ones [28]. These aspects should be considered when comparing our results to previous literature focusing on signal complexity during endurance tasks.

Some study limitations should be acknowledged. As the neuromuscular activity and hormone levels were not evaluated, we do not have information to investigate more in depth the central, peripheral and hormonal responses to WBV applied to isometric endurance tasks. Moreover, our results are limited to this specific isometric endurance assessment and cannot be extended safely to other muscle-contraction modalities. Due to the acute study design, it was not possible to the long-lasting effects of the intermittent isometric endurance task on muscle performance. In addition, as our participants were only males and non-athletes with heterogeneous physical activity levels of a specific age range (26–60 years), our results cannot be generalized to females, athletes, a trained-specific profile or population of other ages. Future studies are needed to address the mechanisms occurring either at central or peripheral level underlying the ineffectiveness of WBV during isometric endurance tasks.

## 5 Conclusion

In the present study, WBV worsened the performance of knee extensors during isometric muscular endurance task in non-regularly trained participants. These results may be partially explained by the lack of a standardized protocol to adopt in non-trained individuals. Indeed, it is still a matter of debate which is the optimal WBV protocol to induce a sufficient training stimulus to determine beneficial adaptations. This acute deleterious effect could facilitate long-term optimisation of physical function through WBV-related adaptations.

## Ethic statement

Participants or their legal guardians signed the written informed consent which was approved by the regional ethical committee (approval number n. 122/2021). All the procedures comply with the Declaration of Helsinki on studies on human participants. Informed consent was obtained from all participants for the publication of all their data and/or images.

## Data availability statement

Dataset will be made available upon request.

## CRediT authorship contribution statement

**Francesca Greco:** Writing – original draft, Methodology, Investigation, Formal analysis, Data curation. **Gian Pietro Emerenziani:** Writing – review & editing, Supervision, Methodology, Funding acquisition, Formal analysis, Data curation, Conceptualization. **Katia Folino:** Investigation. **Marco Spadafora:** Investigation. **Loretta Francesca Cosco:** Investigation. **Carolina Muscoli:** Writing – review & editing, Supervision. **Paolo Sgrò:** Writing – review & editing, Supervision. **Federico Quinzi:** Writing – review & editing, Visualization, Supervision, Investigation, Data curation.

## Declaration of competing interest

The authors declare that they have no known competing financial interests or personal relationships that could have appeared to influence the work reported in this paper.
